# Acute Disseminated Encephalomyelitis following Vaccination against Hepatitis B in a Child: A Case Report and Literature Review

**DOI:** 10.1155/2016/2401809

**Published:** 2016-07-12

**Authors:** Jun-liang Yuan, Shuang-kun Wang, Xiao-juan Guo, Wen-li Hu

**Affiliations:** ^1^Department of Neurology, Beijing Chaoyang Hospital, Capital Medical University, No. 8 South Gongti Road, Beijing 100020, China; ^2^Department of Radiology, Beijing Chaoyang Hospital, Capital Medical University, No. 8 South Gongti Road, Beijing 100020, China

## Abstract

Acute disseminated encephalomyelitis (ADEM) is an inflammatory demyelinating disease of the central nervous system, which has been associated with several vaccines such as rabies, diphtheria-tetanus-polio, smallpox, measles, mumps, rubella, Japanese B encephalitis, pertussis, influenza, and the Hog vaccine. Here, we presented a case of 12-year-old child who suffered from ADEM three weeks after hepatitis B vaccination. He was admitted to our hospital with symptoms of weakness of limbs, high fever, and alteration of consciousness. Some abnormalities were also found in CSF. Treatment with high-dose corticosteroids and intravenous immunoglobulin had significant effect, with marked improvement of the clinical symptoms and the results of CSF. The findings of MRI also detected some abnormal lesions located in both brain and spinal cord. The clinical features, the findings of CSF and MRI, and therapeutic effect may contribute to such diagnosis of ADEM.

## 1. Introduction

Acute disseminated encephalomyelitis (ADEM) is an inflammatory demyelinating disease of the central nervous system (CNS), mainly affecting children and mostly occurring several weeks after infections or more rarely after vaccinations [1]. It involves multifocal areas of the white matter and rarely the grey matter and spinal cord. Effective therapy includes high-dose corticosteroids, intravenous immunoglobulins, and plasmapheresis. The prognosis is generally favourable almost with full recovery [1], although some series have shown 20% mortality, often with high morbidity [2]. A large number of infectious agents or vaccines have been reported to link with ADEM, such as infection of hepatitis A virus [3], hepatitis B virus [4], hepatitis C virus [5], and some vaccinations. However, to the best of our knowledge, rare cases with ADEM after hepatitis B vaccination have been reported in a child [6]. Here, we describe a case of 12-year-old child who suffered from ADEM three weeks after the vaccination of hepatitis B.

## 2. Case Report

A 12-year-old child, male, was admitted to the Department of Neurology in Beijing Chaoyang Hospital. Three weeks before his admission, he received the vaccination of hepatitis B. He was with symptoms of myasthenia of limbs and alteration of consciousness. He also had high fever, with the body temperature maintained between 38°C and 38.5°C. He was with no symptoms of headache, dizziness, nausea, vomit, sphincter dysfunction, and optic neuritis. Four years ago, he had suffered from essential thrombopenia, which relapsed two years ago. He had no history of toxic substance, allergy, operation, trauma, blood transfusion, and inheritance history. He was a full-term baby, with normal vaginal delivery. His mother had suffered from a disease of allergic purpura.

Physical examination on his admission showed that he was with somnolence and uncooperative. His pupils were equal in size and round. The optic nerve was normal. Bilateral light reflexes retained. Muscle strength of limbs grading (II level) was found with hypomyotonia. Sensory tests were uncooperative. Abdominal reflex and cremasteric reflex were negative. The signs of bilateral Babinski and Gordon were positive. Ankle clonus was also detected. Neck resistance was also found.

On admission, the results of blood test were as follows. WBC level (15.8 × 10^9^/L) and the proportion of neutrophils (85.2%) were markedly increased. Procalcitonin was 0.05 ng/mL, which indicated no bacterial infection. C-reactive protein was increased, which was 1.14 mg/dL (0–0.8 mg/dL). ESR was 50 mm/H (2–15 mm/H). IgG was 1750 mg/mL (751–1560 mg/mL). IgA, IgM, C3, and C4 were normal. Sputum cultures of bacteria, fungus, virus, and tuberculosis were negative. Total protein and albumin in blood were decreased. Globulin and total bilirubin were normal. The cerebrospinal fluid (CSF) examinations showed increased pleocytosis (52/*μ*L) and leucocyte count (40/*μ*L). The CSF was composed of 90% mononuclear cells and 10% polynuclear cells. Pandy test was negative. Total protein was normal. Glucose (4.77 mmol/L) was slightly increased (2.5–4.4 mmol/L). Chloride (115.1 mmol/L) was slightly decreased (118–129 mmol/L). Oligoclonal band was found. The aquaporin 4 antibody was negative. Bacteria, mycobacterium tuberculosis, virus (e.g., herpes simplex encephalitis virus, cytomegalovirus, and Epstein-Barr virus), and fungal cultures from CSF and blood serology, with PCR also performed, were negative.

**Figure 1:**
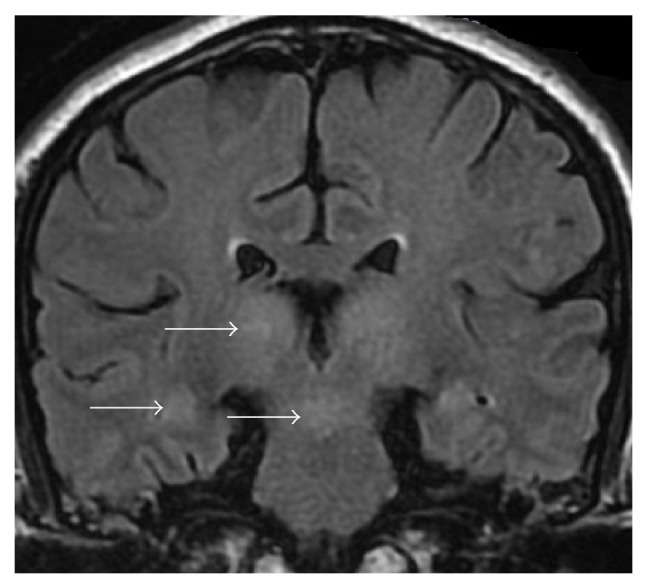
There are widespread abnormal signals at bilateral thalamus and hippocampus on FLAIR image.

**Figure 2:**
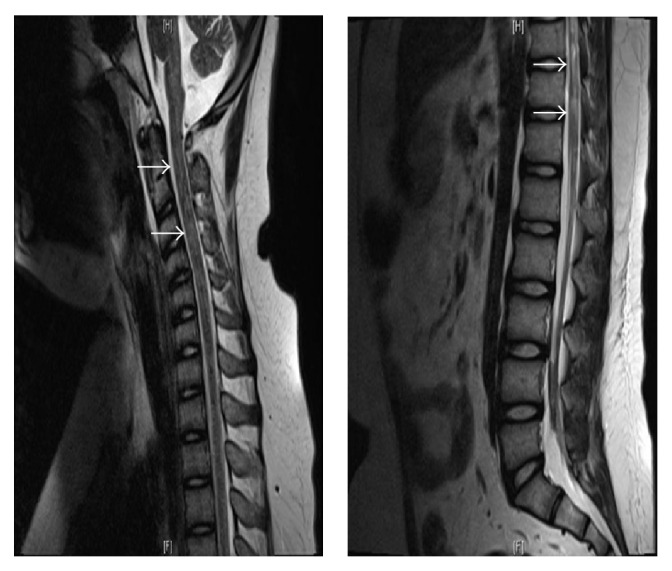
There are extensive lesions in the cervical spinal cord and lumbar spinal on T2 image.

Three days after his admission, MRI of brain and spinal cord also revealed some abnormal findings. His brain MRI showed widespread abnormal signals on FLAIR image (Figure 1). Spinal cord MRI showed that there were abnormal multifocal, strip long T1 and T2 signals at the cervical and intumescentia lumbalis (Figure 2). Furthermore, evoked potential such as brainstem auditory evoked potentials and somatosensory evoked potential also showed some abnormal changes in this case. The visual evoked potential was normal.

 With treatment with high-dose methylprednisolone and intravenous immunoglobulin and also with some antibiotic and antiviral therapy, he showed a dramatic improvement of the clinical and CSF results. About four months later, he recovered completely and there was no relapse during three years of follow-up.

## 3. Discussion

ADEM, a monophasic inflammatory demyelinating disease of the CNS, affects both children and adults and is more frequent in younger people. The incidence of ADEM has been described between 0.4 and 0.8 per 100,000 of population from different causes and pathogenesis [7, 8]. The postinfectious and postvaccination encephalomyelitis may make up about three-quarters of ADEM cases [6]. After prodromal several days or weeks [8], the clinical signs and symptoms usually include altered consciousness and multifocal neurological disturbances. While mild lymphocytic pleocytosis and elevated proteins are detectable in the CSF in ADEM, oligoclonal bands are rarely observed. MRI is considered as a better diagnostic tool of ADEM, with the findings of extensive, multifocal, subcortical white matter abnormalities. Treatment options for ADEM mainly consist of anti-inflammatory and immunosuppressive agents. The prognosis is generally considered to be favourable.

Hepatitis B vaccination has been performed in the national immunization programmes within the last 30 years and is mandatory for newborns and children, especially in developing countries [9]. In spite of its significant contributions of overcoming diseases, previous studies have indicated that hepatitis B vaccination may lead to many demyelinating diseases [10], such as multiple sclerosis, Guillain-Barré syndrome, and acute transverse myelitis. Postvaccination ADEM has been associated with several vaccines such as rabies, diphtheria-tetanus-polio, smallpox, measles, mumps, rubella, Japanese B encephalitis, pertussis, influenza, hepatitis B, and the Hog vaccine. However, the pathogenesis of ADEM following administration of hepatitis B vaccine remains unclear [10, 11]. One crucial theory was “molecular mimicry” hypothesis, which was described as an autoimmune reaction caused by a host receiving an antigen that has amino acids homology with amino acid chains in organs of the host's body. As far as we know, the hypothesis of “molecular mimicry” may be the key pathogenic factors leading to ADEM [12].

In the future, more attention should be paid to patients with the vaccination of hepatitis B when they show some neurological deficits. Despite the rare occurrence of this neurological complication after vaccination, physicians should pay more attention to such rare adverse events. Considering some studies so far do not support a causal relationship between hepatitis B vaccination and MS or other demyelinating diseases, as a result, the relationship between hepatitis B vaccine and CNS demyelinating diseases still needs to be clarified in the future.
